# Ecological Effects on the Dynamics of West Nile Virus and Avian *Plasmodium*: The Importance of Mosquito Communities and Landscape

**DOI:** 10.3390/v13071208

**Published:** 2021-06-23

**Authors:** Martina Ferraguti, Josué Martínez-de la Puente, Jordi Figuerola

**Affiliations:** 1 Department of Theoretical and Computational Ecology (TCE), Institute for Biodiversity and Ecosystem Dynamics (IBED), University of Amsterdam, Science Park 904, 1098XH Amsterdam, The Netherlands; 2 Department of Parasitology, University of Granada, E-18071 Granada, Spain; 3 Doñana Biological Station (EBD-CSIC), E-41092 Seville, Spain; jordi@ebd.csic.es; 4 CIBER of Epidemiology and Public Health (CIBERESP), Spain

**Keywords:** ecology, emerging and remerging diseases, haemosporidians, insect vectors, mosquito-borne pathogens, wildlife

## Abstract

Humans and wildlife are at risk from certain vector-borne diseases such as malaria, dengue, and West Nile and yellow fevers. Factors linked to global change, including habitat alteration, land-use intensification, the spread of alien species, and climate change, are operating on a global scale and affect both the incidence and distribution of many vector-borne diseases. Hence, understanding the drivers that regulate the transmission of pathogens in the wild is of great importance for ecological, evolutionary, health, and economic reasons. In this literature review, we discuss the ecological factors potentially affecting the transmission of two mosquito-borne pathogens circulating naturally between birds and mosquitoes, namely, West Nile virus (WNV) and the avian malaria parasites of the genus *Plasmodium*. Traditionally, the study of pathogen transmission has focused only on vectors or hosts and the interactions between them, while the role of landscape has largely been ignored. However, from an ecological point of view, it is essential not only to study the interaction between each of these organisms but also to understand the environmental scenarios in which these processes take place. We describe here some of the similarities and differences in the transmission of these two pathogens and how research into both systems may facilitate a greater understanding of the dynamics of vector-borne pathogens in the wild.

## 1. Introduction

### 1.1. A General Perspective of Vector-Borne Diseases

Vector-borne diseases (VBD) are illnesses that are caused by a pathogen transmitted to humans or other animals by blood-feeding arthropods such as mosquitoes, ticks, and fleas. Today, VBD account for more than 17% of all infectious diseases and are responsible for over 700,000 deaths annually [1]. One of the most relevant human VBD is malaria, a common parasitic infection caused by protozoa transmitted by anopheline mosquitoes that provokes 219 million infections and causes over 400,000 deaths worldwide every year. Other relevant viral diseases transmitted by vectors include the mosquito-borne diseases Dengue fever, Chikungunya fever, Zika virus fever, Yellow fever, West Nile fever, and Japanese encephalitis, as well as tick-borne encephalitis [1]. 

The incidence of VBD is a worldwide concern for human and wildlife health [2]. Unfortunately, the magnitude of the problem has only recently become apparent given that, for example, the number of annual reports of VBD doubled in the United States in the period 2004–2016 [3]. This changing situation is due in part to factors linked to global change including—but not exclusively so—habitat fragmentation and alteration, land-use intensification, biotic exchange favoring the introduction and establishment of alien species, and climate change operating at a global scale [4]. Indeed, the distribution and incidence of VBD is determined by a complex set of environmental, demographic, and social factors in which global travel and trade and urbanization play key roles.

Humans and wildlife are currently threatened by VBD [5,6]. Most emerging infectious diseases affecting humans are of zoonotic origin and are maintained in the wild in populations of non-human vertebrates [7,8]. Hence, understanding the factors that regulate the transmission of pathogens in the wild is of great importance for ecological, evolutionary, health, and economic reasons. Pathogens are biological agents belonging to a number of very different taxonomic groups that include viruses, protozoa, and helminths, harbored by a host and harm its health, although the level of virulence differs widely in the studied host–pathogen assemblages. Both pathogens and hosts are in constant competition for resources, and selection pressure on the former favors their development and transmission rates, while pressure on the latter favors strategies—e.g., physiological, and behavioral responses—that prevent infection or minimize its cost [9]. Pathogens play a key role in regulating the size and dynamics of host populations and are important selective factors in their hosts [10]. In the case of zoonotic pathogens transmitted by insect vectors, the life cycles of pathogens involve several phases and stages of development that alternate between different host groups, from invertebrates (e.g., insects) to vertebrates.

The transmission of vector-borne zoonotic pathogens entails the interaction of these organisms with their insect vectors and vertebrate hosts (i.e., wildlife, livestock, or humans), a process that is greatly influenced by environmental factors including climate but also habitat characteristics [11]. Traditionally, the study of pathogen transmission has focused on just one or two of these factors, and the remaining ones have tended to be ignored. However, from an ecological point of view, it is essential not only to study the interaction between these organisms but also to understand the environmental context in which these processes take place. Thus, the study of VBD requires an integrative approach that combines knowledge of the pathogens circulating between the communities of both vectors (e.g., mosquitoes) and vertebrate hosts, along with an understanding of the environmental characteristics potentially affecting these interactions. Here, we explore the transmission dynamics and the factors potentially affecting two VBD—West Nile virus (WNV) and the avian malaria parasites of the genus *Plasmodium*—that circulate naturally between birds and mosquitoes. In this literature review, we highlight the similarities and differences between their host–vector–parasite systems and describe how research can provide useful information for advancing the understanding of how biotic and abiotic environmental factors can affect the transmission of these vector-borne pathogens.

### 1.2. West Nile Virus and Avian Plasmodium Parasites

West Nile virus is a generalist virus belonging to the genus *Flavivirus* (family Flaviviridae), with a complex eco-epidemiology, which is today regarded as an emerging zoonotic arbovirus (arthropod-borne virus). Historically, the range of WNV embraced Eurasia and Africa, continents in which there have been numerous outbreaks over the past 10 years [12]. Today, however, WNV is widely distributed worldwide and is present in Europe, America, Africa, the Middle East, Western Russia, Southeast Asia, and Australia. Based on genomic phylogeny, at least seven WNV lineages have been identified [13], the commonest being lineage 1.

Lineage 1 was dominant in the twentieth century in Europe [13] and is dominant today in Africa [14], Asia [15], and, since 1999, America [16]. In the USA, WNV chiefly affects birds and humans, and 51,801 WNV cases have been reported in humans, leading to 2390 deaths (Available online: https://www.cdc.gov/westnile/statsmaps/cumMapsData.html#four (accessed on 22 June 2021)). By contrast, WNV lineage 2 was originally restricted to sub-Saharan Africa but was detected for the first time in Europe in Austria in 2004 and has spread since then to other countries including Greece, Hungary, Italy, Russia [17,18], and Spain, where it was detected in a northern goshawk (*Accipiter gentilis*) in Catalonia [19]. Moreover, a putative novel lineage infecting wild-collected mosquitoes was detected in Spain in 2006 [20]. Curiously, unlike other WNV lineages, lineage 4 has been detected in frogs as well as mosquitoes [13,21].

**Figure 1:**
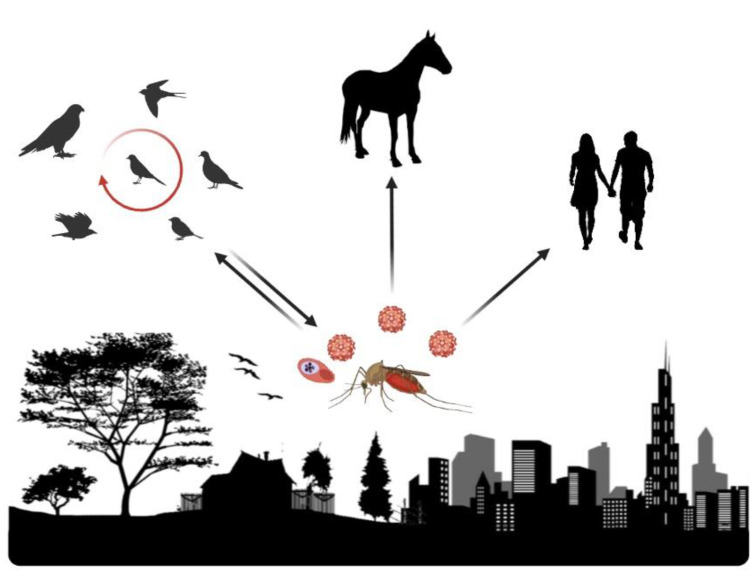
The life cycle of WNV and avian malaria parasites. For WNV, the primary transmission cycle takes place between mosquitoes and birds. Some infected birds develop high levels of viremia in their bloodstreams, and mosquitoes become infected after biting these infected birds. Afterwards, the infected mosquito bites other birds and transmits the virus (primary transmission cycle) or, alternatively, bites and infects people, horses, or other mammals (incidental hosts). The avian *Plasmodium* transmission cycle is maintained exclusively between birds and mosquitoes. Image created with ©BioRender.

West Nile virus is a multi-host, multi-vector pathogen with the ability to replicate in many species of vertebrates and mosquitoes. Indeed, WNV efficiently replicates in more than 300 species of birds (Available online: https://www.cdc.gov/westnile/dead-birds/index.html (accessed on 22 June 2021)), and viremias are reached that are high enough to infect the various mosquito species through which it is transmitted [22]. Thus, most competent hosts for WNV transmission are birds [23,24], although some amphibians, reptiles, and mammals (e.g., squirrels (*Sciurus* sp.), eastern chipmunks (*Tamias striatus*), and eastern cottontail rabbits (*Sylvilagus floridanus*)) are also known to be competent hosts [25,26]. West Nile virus infects horses and humans via mosquito bites, but they are accidental dead-end hosts, i.e., they are not competent hosts, although they can suffer from WNV-associated diseases (Figure 1) [27,28].

In Europe, birds do not usually show any clinical signs or mortality [29], and WNV infection occurs asymptomatically for 5–10 days post-infection [24]. However, some avian groups including gulls, rallids [30], raptors [31], and crows [32] frequently have higher prevalence of WNV antibodies ([33], see also [24] and references therein), and cases of mortality are reported in some species [34,35,36]. Given the short duration of the viremia, exposure to WNV is normally established via the detection of antibodies [24]. In humans, WNV infection is usually asymptomatic, although approximately 20% of infected people develop a flu-like illness. In a very low percentage (less than 1%), however, infection can severely affect the nervous system and is lethal in approximately 10% of severe cases [37]. Likewise, most WNV infections are asymptomatic in horses, and only 10% of infected animals suffer any neurological symptoms of the disease, which is lethal in approximately one third of all symptomatic cases [38]. Nevertheless, in recent years, the virus has significantly expanded its incidence and range, with 624 and 148 autochthonous cases diagnosed in humans in 2010–2015 in Greece and Italy, respectively [39]. Particularly alarming was the enormous increase in cases during 2018, when over 1500 human cases and 180 deaths were reported in Europe—seven times the number of infections detected in 2017 [40]—with incidences in human populations that differed greatly between countries. More recently, in 2020, 40 confirmed and 37 probable cases of human WNV were reported in Spain, leading to 7 deaths [41].

Haemosporidians (Sporozoa: Haemosporidia) including malarial parasites are blood parasites that infect amphibians, reptiles, mammals, and, above all, birds and use hematophagous dipterans as vectors [42]. Avian *Plasmodium* infections are an important concern in poultry [43] and certain bird species such as penguins that are kept in captivity [44]. The introduction of avian *Plasmodium* in areas inhabited by immunologically naïve species has contributed notably to the decline of certain bird populations [45], and *Plasmodium relictum* is considered to be one of the 100 worst invasive Haemosporidian species [46]. In addition to their relevance to conservation, the ecological and evolutionary consequences for their vertebrate hosts of infection by these parasites have been studied [47,48,49,50,51]. Avian *Plasmodium* are transmitted by different species of mosquitoes [42,52]. Parasites usually persist in birds for many years and produce chronic infections that are a potential source of infection for vectors for as long as the bird lives [42]. Although some studies have reported the apparent disappearance of parasites in the bloodstream of repeatedly sampled birds [53], the chronic infections caused by the avian malaria parasite clearly contrast with the usually short-lived WNV viraemia in blood [54]. This explains the different methodologies based on detecting WNV antibodies in birds [55,56] but on parasite DNA in the case of malarial parasites [57,58]. Nevertheless, serological approaches have also been used to identify anti-*Plasmodium* antibodies in birds [59,60]. The use of these two methodologies to study avian malaria parasites allows researchers to obtain a general overview of the immunological responses of birds to parasite infection and to study the tolerance and resistance of birds to infection by *Plasmodium*. For WNV, studies focusing on the detection of viral RNA in birds usually focus on birds showing clear symptoms of the disease since the short duration of the viraemia in the bloodstream makes the screening of apparently healthy birds somewhat impractical.

Despite their clear epidemiological differences, WNV and *Plasmodium* have important similarities in their transmission routes. For example, both pathogens require a competent mosquito vector to be transmitted from an infected bird to a new host. Although they are regarded as multi-vector pathogens, only some mosquito species are competent for transmitting these pathogens efficiently. WNV replicates mainly in mosquitoes of the genus *Culex* (Diptera: Culicidae) and, above all, in the ornithophilic species (e.g., *Culex modestus*, *Cx. perexiguus*, *Cx. pipiens*) that are the main vectors of WNV in Europe [61,62]. WNV has also been detected under natural conditions in other mosquito species such as *Aedes* (*Ochlerotatus*) *caspius*, although their competence as vectors of WNV is still under debate [63], and experimental infections in the laboratory have confirmed that this species’ vector competence is less than that of the *Culex* mosquito species [62]. On the other hand, *Plasmodium* is regarded as a very generalist parasite that can be transmitted by mosquitoes of the genera *Culex*, *Aedes*, *Anopheles*, and *Culiseta* [64,65], and it is likely that *Cx*. *pipiens* plays a central role in this transmission [66]. This aspect of *Plasmodium* transmission is an expanding area of research. For instance, the use of molecular xenomonitoring, a surveillance technique that involves the collection and testing of insect vectors to identify pathogen DNA and RNA (i.e., their genetic material) that is highly relevant to human and animal health, has been applied to the study of mosquitoes and has provided valuable information on the potential vectors of *Plasmodium* in the wild [67,68,69]. The use of molecular techniques has revealed the presence of *Plasmodium* DNA in many different species of mosquitoes [65]. However, the identification of parasite DNA in mosquitoes does not demonstrate vector competence, as the identification of abortive forms of the parasites may occur [70]. For example, although the presence of parasite DNA has been reported in mosquitoes such as *Ae*. *caspius* [67], the experimental exposure of *Ae. caspius* to the blood of infected house sparrows demonstrated their extremely low vector competence for *Plasmodium* since none of these mosquitoes had parasites in their saliva 13 days post-exposure [71]. Consequently, experimental studies of the vector competence of a wide range of mosquitoes are still necessary for a fuller understanding of the nature of vector–parasite interactions in natural communities.

Although work on these pathogens under natural conditions has been performed in Europe and America [12,13], studies that combine information on both pathogens are still few and far between. To our knowledge, only a handful of studies have ever identified the frequency of coinfections by WVN and avian *Plasmodium* in birds. Avian *Plasmodium* infections and the presence of WNV antibodies in adult birds were found to be negatively associated in the study by Medeiros et al. [72] conducted in a bird community in Chicago. However, a similar prevalence of *Plasmodium* infection was found in passerines with and without WNV antibodies in Spain [66]. Studies of the related mosquito-borne flavivirus USUTU virus (USUV) found a high rate of coinfection by avian *Plasmodium* and USUV associated with bird mortality in the Netherlands [73] and Belgium [74]. Mixed infections are expected to be more virulent for birds than single ones [75], and so, avian *Plasmodium* could have fatal consequences in cases of infection by flaviviruses [73]. This possibility merits further research since experimental evidence is lacking and the mechanisms clarifying these associations are still unknown (e.g., immune responses). In addition, one of the explanations for coinfection is that both pathogens can be transmitted at the same time by the same mosquitos. Medeiros et al. [76] identified the presence of WNV and avian *Plasmodium* in mosquito pools in Chicago and found that WNV infection was positively related to *Plasmodium* infection. However, given that in this case, the data for mosquitoes were pooled, it is unclear whether or not the same or different individuals harbored both pathogens. Nevertheless, these results suggest that due to their similar ecology, both pathogens co-circulate in the same vectors (i.e., *Culex pipiens*/*restuans*) in this area.

## 2. Effects of Landscape Change on West Nile Virus and Avian *Plasmodium*

Global change is expected to affect VBD distribution and incidence given that the environment plays an important role in vector-borne pathogen transmission [4,11], probably through its effects on vector and host populations [77] and on pathogens´ development in vectors [78,79]. This may be the case for pathogens such as avian malaria parasites that have been introduced into new areas and have had a serious negative impact on native bird populations [45]. It has been suggested that *Plasmodium* prevalence has also increased in recent decades due to climate change, especially in parts of Africa and Europe [80]. However, most of these effects have yet to be empirically tested [81,82]. Forest management and fragmentation, urbanization, and agricultural expansion have all been linked to increases in the incidence of several infectious diseases, including Lyme borreliosis (*Borrelia*) in the northeast United States [83], Nipah virus in Malaysia [84], and human malaria [8,85,86]. This is also the case of the pathogens—e.g., avian malaria parasites [87,88]—that infect birds, probably due to the effect of changes in land use on insect vector distribution and abundance [89]. In fact, two of the main West Nile virus vectors in Europe are uncommon in built-up areas [90]. *Cx. modestus* is mainly found in brackish marshes and flooded paddy fields [91] and breeds preferentially in rice fields [90], thereby confirming the potential of rice paddies to act as factors in mosquito-borne disease transmission [90]. Indeed, the presence of *Cx. modestus* mosquitoes has been historically associated with WNV outbreaks in rice-growing areas in the Camargue region in France [92]. Likewise, *Cx. perexiguus*, a key bridge species in the epizootic transmission of WNV to horses in southern Spain [61], is abundant in stagnant and ephemeral swamps, streams, and paddy fields [90,91], all typical breeding habitats for many host bird species, which thus may provoke the zoonotic WNV amplification cycle and the spread of avian malaria parasites. Finally, *Cx. pipiens* is the most common mosquito species in urban areas where it uses water bodies such as vases in cemeteries, clay flowerpots, storm drains, or puddles for oviposition, and larvae are frequently found in artificial water bodies [91]. It has been suggested that this species plays a key role in the epizootic transmission of WNV to humans in built-up areas [93,94] and of *Plasmodium* to birds [66].

Human-mediated landscape disturbance influences the dynamics of wildlife diseases by affecting the distribution of both host [95,96] and mosquito species [97]. Due to their respective ecologies, insects and birds are affected by changing environments in different ways. Human activities including animal husbandry and intensive agriculture may also affect the transmission of VBD directly or indirectly though their impact on water availability or on the occurrence and composition of competent vectors and hosts [4,98]. Factors such as vegetation cover [99] and water sources [100] affect vector and/or host communities and, ultimately, determine the risk of pathogen transmission. Different types of forests and their management and structure are related to avian malaria prevalence [82,88,89,101,102,103]. However, reported patterns differ between studies. For example, Bonneaud et al. [104] showed that the prevalence of *Plasmodium* parasites in birds was higher in pristine than in deforested areas of Cameroon. By contrast, the prevalence of avian malaria parasites in the olive sunbird (*Cyanomitra olivacea*) in Ghana was highest in secondary forests with lower levels of disturbance (i.e., deforested areas) [81]. Moreover, the diversity and prevalence of avian *Plasmodium* was found to increase in disturbed habitats in two widespread species of African rainforest birds [101]. A recent study by Reis et al. [105] also reported that the prevalence of *Plasmodium* was related to land use, with the highest infection prevalence occurring in anthropogenic areas including open areas and shade plantations. Additionally, Hernández-Lara et al. [88] noted a lower prevalence of *Plasmodium* in birds from a well-preserved forest and a coffee plantation than in a periurban forest cattle ranch and urban greenspace in Veracruz, Mexico. Parasite prevalence may be also related to habitat structure. The Lesser Antillean bullfinch (*Loxigilla noctis*), a common passerine endemic to the Caribbean, had greater *Plasmodium* prevalence in highly fragmented habitats, and forest fragmentation was found to be more important than forest loss in predicting greater parasite infection [106]. Additionally, birds from paddy fields (rural agricultural areas) tend to have higher haemosporidian prevalence than birds from more anthropized areas [107]. This disparity in results probably reflects the varying conditions that different habitats provide for mosquitoes to breed and indicates the effects that habitat fragmentation and/or transformation have on the abundance and composition of vector communities. In addition to parasite prevalence, a number of studies with contrasting results have investigated potential differences in the intensity of infection in birds from different habitat types. For instance, in the case of parasite prevalence, parasitemia in olive sunbirds was higher in better conserved areas than in deforested habitats [76]. Conversely, the intensity of infection did not vary between different types of land uses (i.e., preserved montane cloud forests, coffee plantations, cattle ranches, periurban forests, and urban greenspaces) [88] or between forests with different management systems [108]. However, it is important to note that parasite prevalence and parasitemia may reflect different aspects of parasite infections, with the latter being more likely to be linked to host-related factors (e.g., immunocompetence) than to habitat characteristics and their effects on vector abundance. A number of factors including parental effort are known to affect the intensity of infection in birds, although there are few studies of this question in relation to WNV. This fact may affect—at least in part—the transmission dynamics of pathogens since the intensity of infection in vertebrate hosts could determine the success of pathogen development in vectors ([109], but see [110]).

Studies of WNV also provide evidence suggesting that its distribution could be linked to socio-demographic factors influencing vector and host ecology and, thus, the potential disease distribution (e.g., Ruiz et al. [111]). These authors found a relationship between WNV and urban environments whereby the age of the housing, land use, and concomitant social and natural features can influence the transmission of the vector-borne virus in cities. The environmental and ecological drivers of WNV transmission are complex and are not as yet known in detail. Indeed, many environmental factors including human activities may boost the population density of mosquito vectors. Examples of these factors include irrigation and heavy rains followed by floods and then warm, dry weather, and higher-than-usual temperatures [112]. Changes in land use are expected to affect the dynamics of WNV transmission. Different studies conducted in the USA support this possibility, and, for example, the probability of finding WNV-antibody positive birds increased from urban to suburban land use with moderate housing density in Georgia, probably due to different vector habitat preferences [113]. In addition, WNV seroprevalence rates were higher in two suburban areas than in two rural ones in Louisiana [114]. By contrast, WNV seroprevalence was higher in birds from urban areas than in those from natural areas in Illinois [115]. In the northeast United States, WNV disease incidence was found to be highest in urban areas with intermediate forest cover [116]. Thus, as in the case of avian malaria parasites, habitat characteristics are clearly associated with WNV disease dynamics and ecology, probably due by their effects on host and vector presence, behavior, and interactions. Finally, the relationship between climate and mosquitoes is widely accepted to be another key factor since seasonal changes strongly dictate mosquito population dynamics and virus–mosquito transmission efficiency [117,118,119,120,121]. Temperature affects WNV transmission through its effect on mosquito reproduction rates and biting behavior [122,123] as well as on the extrinsic incubation period of the virus in the vectors [124]. The importance of temperature for both *Culex* mosquito dynamics and WNV circulation has been recognized by several studies conducted in Europe (e.g., [123,125,126,127]) and North America (e.g., [128,129,130]). Indeed, temperature and temperature fluctuations significantly affect—albeit not always linearly—mosquito abundance, biology, and physiology. For instance, in coastal and inland areas of Spain, the annual abundances of *Cx. pipiens*, a common vector of WNV, are affected by changes in temperature and rainfall patterns [131,132], although no changes in the abundance of this mosquito species in southern Spain are expected under different climate change scenarios as discussed by Roiz et al. [131], because of the opposed changes in temperature and rainfall expected in the area. These authors found that the relationship with temperature was not linear and that *Cx. pipiens* abundances were lower in very hot years than in years with more moderate summer temperatures. Climate change may favor the establishment and endemic circulation of WNV in southern Europe, western Asia, the eastern Mediterranean, the Canadian Prairies, and parts of the USA and Australia [133]. Furthermore, in Canada, WNV is spreading largely due to the geographic expansion of its vector *Culex tarsalis*, presumably because of global warming [134]. Similarly, climate change is also expected to affect the spread and transmission dynamics of avian malaria parasites [80,135].

In addition, human activities may also have an impact on pathogen–mosquito interactions due to their effect on the capacity of mosquitoes to transmit pathogens. For example, the interactions between pathogens and mosquitoes may be determined by the presence of pollutants derived from human activities. *Culex pipiens* larvae fed a standard diet had higher avian *Plasmodium* prevalence when exposed to the herbicide glyphosate [136]. In addition, antibiotics, and other personal care products in waters in which mosquitoes breed may affect mosquito development and the epidemiology of mosquito-borne pathogens [137]. In particular, exposure to antibiotics affects the survival cost and transmission rates of avian malaria parasites by mosquitoes [138] and may also affect the dynamics of the transmission of arbovirus [137].

## 3. Pathogen Prevalence and Mosquito Community Composition

Vector communities affect pathogen amplification and will ultimately determine the incidence of VBD in vertebrate hosts [77,139,140]. Mosquito species differ in their climatic and habitat requirements, feeding preferences, and vector competence, which is likely to have an effect on the epidemiology of VBD, including WNV and avian malaria parasites. Anthropogenic landscape transformation often leads to an increase in the abundance of just a few species and a general loss of biodiversity [141], especially in urban areas [95]. These processes may alter the transmission of vector-borne pathogens due to their impact on mosquito abundance and distribution [90,142].

A variety of factors including the presence of artificial habitats such as water deposits, gardens, and underground water systems that provide alternative breeding sites for mosquitoes are key drivers of the impact of urbanization on vector populations. Some species of the genera *Culex* [143], *Aedes* [144], and *Anopheles* [145] are in fact favored by urbanization. For instance, a recent distribution model of the avian malaria vector *Cx. pipiens* directly and positively linked the presence of this species to the degree of urbanization [146]. The density, as well as the larval development and adult survival rates, of the invasive mosquito *Aedes albopictus* could be greater in built-up areas [147]. The asymmetric competition between larvae of this invasive species with *Cx. pipiens* may affect *Cx. pipiens* populations where these species live in sympatry [148,149,150]. By contrast, the abundances of other mosquito species decrease along a natural–rural–urban gradient [90,151], thereby reducing the potential for pathogen transmission. This is the case of WNV infection rates in *Culex* mosquitoes, which declined with greater wetland cover in Louisiana [152], even though the wetland surface area was not significantly associated with either vector density or host community abundance and composition. On the other hand, the percentage of wetland cover was highly related to host community composition, which suggests that the possible effect of wetland areas was mediated by the impact of host community composition on the WNV infection risk [152]. Overall, these results support contrasting patterns that are determined by pathogen identity, the infection parameter measured (incidence in the vector or vertebrate host), and landscape characteristics, all of which indicate that local patterns have an impact on the community of vectors and, ultimately, will define the transmission dynamics of vector-borne pathogens in the area. 

In addition to host and vector species composition and abundance, mosquito host selection has been identified as a key factor modulating the amplification and transmission success of WNV [28]. Mosquito species differ in their feeding preferences, with some species biting mainly mammals and others preferring birds as hosts [153]. Obviously, this differential feeding behavior will determine the contact rates between mosquitoes and infected/susceptible vertebrates and hence modify the risk of pathogen transmission [28,61]. Some mosquito species facilitate the transmission between reservoir hosts, while others act as bridge vectors between infected competent hosts and susceptible hosts [61,154]. This is particularly relevant in the case of pathogens circulating between birds, which occasionally infect mammals and cause diseases, as in the case of WNV. The effects of urbanization on the feeding patterns of mosquitoes have been tested for some species and have thrown up contrasting results. For example, in Italy, humans dominated the diet of *Ae. albopictus* in urban areas and represented 79–96% of bloodmeals, a percentage that decreased to 23–55% in mosquitoes from rural areas, where horses and bovines were the most bitten hosts [155]. On the other hand, in a study conducted in the United States, Faraji et al. [156] found that *Ae. albopictus* fed more on humans in suburban than in urban areas. In the case of *Cx. pipiens*, a major vector of avian malaria parasites and WNV, birds were identified as the main feeding source, while in southern Spain, a similar percentage of bird-derived bloodmeals was found in urban, rural, and natural areas [157]. These differential feeding patterns between species undoubtedly affect the exposure of mosquito species to the pathogens circulating in birds. The identification of avian malaria parasites in mosquito bloodmeals revealed a higher prevalence in *Cx. pipiens* than in *Ae. albopictus* trapped in Italy [158], which further supports the differential relevance of these species in the transmission of avian pathogens [159,160]. 

In any study of VBD, the vector competence of each mosquito species must also be taken into account [161]. Given that not all mosquito species are competent vectors for all the pathogens they interact with [70,162], different hematophagous insect species will play differing roles in the transmission cycles of each pathogen [30,163,164]. A recently published epidemiological model found evidence for the differential contribution of several mosquito species in the transmission of avian *Plasmodium* and WNV [66]. By estimating the basic reproductive number *R_0_* in areas with different mosquito species, in this study, we concluded that *Cx. perexiguus* was the most relevant species in the amplification of WNV in southern Spain. By contrast, *Plasmodium R_0_* values were higher when *Cx. pipiens* was present in the population, either alone or in combination with other mosquito species such as *Cx. modestus* or *Cx. perexiguus*. These results suggest that pathogen transmission has different spatial and temporal patterns associated with different vectors, thereby highlighting the importance of considering the overall composition of the insect community, mosquito feeding patterns, and vector competence when studying VBD transmission [66]. However, our study was limited by the fact that it did not include a wide range of potential reservoir hosts for WNV. In addition, most of these types of studies are hampered by the absence of basic information on the competence of mosquito species for transmitting each pathogen species or lineage/strain, which will have an important effect on their epidemiology [71,165].

Epidemiological and mathematical modeling approaches can provide essential tools for understanding the transmission dynamics of pathogens and so allow us to compare and evaluate different prevention and control measures, a task that would otherwise be nonviable using only data-driven models [166]. Emerging zoonotic VBD often have a wide range of vertebrate reservoirs and several vector species, and future models need to incorporate this complexity and its relevance to pathogen amplification. This is also relevant for the case of parasites such as avian *Plasmodium*, and despite the existence of multi-host species able to infect different species, clear differences in host ranges between parasite lineages have been identified [167,168]. Consequently, these parasites provide a suitable model for empirical and theoretical analyses of the relationships between biodiversity, host–vector range, and pathogen amplification. Although these areas of knowledge might initially appear to be highly disparate, they all have the same common denominator under the One Health approach. Thus, by integrating ecology into public and animal health research, we will be able to improve our understanding of the epidemiology and the risk of transmission of mosquito-borne pathogens.

Simultaneously studying WNV and other mosquito-borne pathogens such as *Plasmodium* in the same localities is a very powerful tool for separating the impact that vector and host communities have on pathogen amplification. This is because the same community composition will provide different capacities for the amplification and transmission of each pathogen, which thus signals that any relationship between host and/or vector biodiversity and pathogen prevalence will depend heavily on host community composition and pathogen characteristics [169,170].

## 4. Conclusions

Avian *Plasmodium* and WNV share certain ecological aspects that clearly determine their epidemiology. However, all too often, research projects focus only on certain aspects of their ecology, and, for instance, vector competence is more studied in WNV, whereas the impact of landscape is more studied in *Plasmodium*. Good evidence exists to support the role of habitat on infection patterns in wild birds, which are probably driven, at least in part, by the impact of landscape on the vector community. This would ultimately determine the epidemiology of vector-borne diseases, even if to date, the results of published analyses differ according to the pathogen and environmental characteristics studied. Despite their differences, the study of one of these pathogens could provide valuable information for improving understanding of the epidemiology of the others and, more importantly, for comprehending how host and vector communities affect pathogen amplification and transmission to different vertebrate species. Moreover, simultaneously studying pathogens relying on different competent vector and host species could greatly enhance our capacity for understanding the impact of the environment and ecology on pathogen transmission. To date, studies focusing on both WNV and *Plasmodium* are scarce, and so we encourage authors to screen for both pathogens in avian hosts and mosquito vectors, especially in view of the fact that similar vector species are involved in their transmission. The expansion of WNV lineage 1 into the western hemisphere and lineage 2 into Europe, together with the global distribution of avian *Plasmodium*, are enough reasons for examining and attempting to understand the potential interactions affecting the transmission of these pathogens under natural conditions.
